# Visualizing adverse events in clinical trials using correspondence analysis with R-package visae

**DOI:** 10.1186/s12874-021-01368-w

**Published:** 2021-11-09

**Authors:** Márcio A. Diniz, Gillian Gresham, Sungjin Kim, Michael Luu, N. Lynn Henry, Mourad Tighiouart, Greg Yothers, Patricia A. Ganz, André Rogatko

**Affiliations:** 1 Samuel Oschin Comprehensive Cancer Center, Cedars-Sinai Medical Center, Los Angeles, CA USA; 2 Rogel Cancer Center, University of Michigan, Ann Arbor, MI USA; 3 Graduate School of Public Health, University of Pittsburgh and NRG Oncology, Pittsburgh, PA USA; 4 Jonsson Comprehensive Cancer Center and Fielding School of Public Health, University of California, Los Angeles, CA USA

**Keywords:** Data visualization, Correspondence analysis, Adverse event, CTCAE, Clinical trials

## Abstract

**Background:**

Graphical displays and data visualization are essential components of statistical analysis that can lead to improved understanding of clinical trial adverse event (AE) data. Correspondence analysis (CA) has been introduced decades ago as a multivariate technique that can communicate AE contingency tables using two-dimensional plots, while quantifying the loss of information as other dimension reduction techniques such as principal components and factor analysis.

**Methods:**

We propose the application of stacked CA using contribution biplots as a tool to explore differences in AE data among treatments in clinical trials. We defined five levels of refinement for the analysis based on data derived from the Common Terminology Criteria for Adverse Events (CTCAE) grades, domains, terms and their combinations. In addition, we developed a Shiny app built in an R-package, visae, publicly available on Comprehensive R Archive Network (CRAN), to interactively investigate CA configurations based on the contribution to the explained variance and relative frequency of AEs. Data from two randomized controlled trials (RCT) were used to illustrate the proposed methods: NSABP R-04, a neoadjuvant rectal 2 × 2 factorial trial comparing radiation therapy with either capecitabine (Cape) or 5-fluorouracil (5-FU) alone with or without oxaliplatin (Oxa), and NSABP B-35, a double-blind RCT comparing tamoxifen to anastrozole in postmenopausal women with hormone-positive ductal carcinoma in situ.

**Results:**

In the R04 trial (*n* = 1308), CA biplots displayed the discrepancies between single agent treatments and their combinations with Oxa at all levels of AE classes, such that these discrepancies were responsible for the largest portion of the explained variability among treatments. In addition, an interaction effect when adding Oxa to Cape/5-FU was identified when the distance between Cape+Oxa and 5-FU + Oxa was observed to be larger than the distance between 5-FU and Cape, with Cape+Oxa and 5-FU + Oxa in different quadrants of the CA biplots. In the B35 trial (*n* = 3009), CA biplots showed different patterns for non-adherent Anastrozole and Tamoxifen compared with their adherent counterparts.

**Conclusion:**

CA with contribution biplot is an effective tool that can be used to summarize AE data in a two-dimensional display while minimizing the loss of information and interpretation.

**Supplementary Information:**

The online version contains supplementary material available at 10.1186/s12874-021-01368-w.

## Background

The understanding of adverse events is paramount in the assessment of therapies in clinical trials. In the endeavor to support investigators in the challenging task of identifying and documenting toxicities, the National Cancer Institute has maintained, since 1983, an empirical lexicon of AE terms that are commonly encountered in oncology: the CTCAE [1], which has been broadly adopted over the last decades. The criteria classifies AE into 26 domain organ classes and severity grades, such that grade 1 corresponds to a mild or asymptomatic symptom and grade 5 indicates death.

Although such comprehensive criteria has allowed investigators to collect a large amount of AE clinical trial data, the abundance of information has often been ignored in the analysis of clinical trials. Analyzing AE data is a complex task because each patient could experience more than one AE term from different organ domains and different grades of the same AE term during several cycles of treatment resulting in a high-dimensional toxicity profile. Investigators usually present lengthy and overwhelming AE tables, or partial toxicity profiles by treatment after summarizing AE chosen based either on a frequency threshold, severity of AEs or relatedness to treatments [2] into their maximum grade. However, the use of maximum grade leads to loss of information and has been largely criticized and other alternative approaches have been discussed in the literature that summarizes toxicity profiles into a more comprehensive score [3–5].

The CONSORT extension for reporting harm outcomes [6] describes the importance of graphical displays for summarizing AE data. Several graphical summary approaches have been developed in the literature since then: Amit et al. [7] considered dot-plots for the percentage of AE terms’ occurrence by treatment ordered by their relative risks; Zink et al. [8] proposed volcano plots with bubble size proportional to the frequency of AE domain or terms; Thanarajasingam et al. [4] recommended profile plots to illustrate the average toxicity as function of cycle for a given AE term; Karpefors and Weatherall [9] suggested tendril plots to represent the occurrence of a given AE term over time, and Gresham et al. [5] proposed stacked barplots for the AE frequency as function of the number of toxicities per patient and grade toxicity. However, the majority of these approaches cannot be applied to more than two treatments.

Surprisingly, none of the aforementioned approaches have used any traditional statistical high dimension reduction technique such as CA, which is a multivariate technique with the purpose to communicate contingency tables using two-dimensional graphical displays, while quantifying the loss of information. Initial applications of CA and its variants (stacked, multiple, detrended) were broadly discussed in Greenacre [10], with specific applications in epidemiology [11–13] and bioinformatics [14–16].

In this article, we propose the use of stacked CA as a visualization tool for AE data to unravel differences in treatment profiles when comparing their AEs as a complementary tool to toxicity scores [5, 17]. An R-package was developed to make our approach available interactively. We illustrate the use of CA to identify different toxicity profiles among treatments in two clinical trials R04 [18, 19] and B35 [20–24]. Moreover, we apply CA using contribution biplots [25] that are not widely disseminated yet, even though they address long-standing interpretation issues of CA such as outlier points with low contribution to the variance.

## Methods

### Correspondence analysis

The seminal ideas of CA was proposed by Herman Otto Hartley (Hirschfeld) [26], later developed by Jean-Paul Benźecri [27] and disseminated by Michael Greenacre [28, 29]. We will briefly review the main concepts of CA. A detailed mathematical is available as an Additional file.

The goal of CA is to graphically represent contingency tables. Following Greenacre [29], we will apply CA on stacked tables such as Table 1, where *π*_*ij*_ is the relative frequency of *AE*_*i*_ class for treatment *Tj* such that AE classes can be based on three levels of data aggregation: (a) AE grades, (b) AE domains, (c) AE terms and their combinations. While toxicity profiles can be presented and compared based on tables when AE classes are only defined by AE grades, it is a much more complex task when AE classes are defined by AE domains or AE terms, and their combinations with AE grades. There are 26 domains and 790 AE terms in CTCAE v4, which can generate 130 AE classes when AE domains are combined with AE grades and 3950 AE classes when AE terms are combined with AE grades.

**Table 1:**
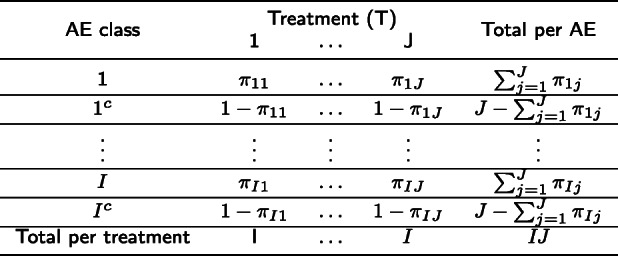
Contingency table 2*I × J* with row and column marginals

The interpretation of Table 1 is asymmetric: we are interested in studying the differences in toxicity profiles among treatments that lie in a high-dimensional space, which will be denoted as toxicity space. Visualizing the toxicity profiles in the toxicity space can give us insight regarding the association between treatments and AE classes. Nonetheless, it is not always feasible to display toxicity profiles when the number of dimensions is greater than three, i.e., four treatment arms (*J ≥* 4) or four AE classes (*I ≥* 4). Moreover, distances between toxicity profiles of treatments are not simple to be evaluated even in a three dimensional space.

In this context, CA seeks the two-dimensional display that minimizes the loss of information when reducing the dimension of the toxicity space. Information is measured through variability, denoted as total inertia in CA, among toxicity profiles of treatments. Asymmetric contribution biplots [30] are two-dimensional diplaying showing the projection of the two dimensions with highest variability in the toxicity space. The first dimension of the biplot represents the direction with highest variability of the toxicity space and the second dimension corresponds to the direction with the second highest inertia. Adding up the inertia of the remaining dimensions allows us to quantify the loss of information. Therefore, we can evaluate whether the two-dimensional representation of the toxicity space is adequate.

The inertia associated with each dimension can also be broken down based on the contributions of each AE class. Dimensions can be interpreted based on AE classes with high contributions. AE classes with high contributions to a dimension ares’ identified based on their distance from the origin in the same direction of that given dimension. Then, a treatment with high frequency of a given AE class will have high values in the same dimension and direction of that AE class.

In this way, we are able to compare toxicity profiles of treatments as following:

1. Interpret each dimension based on the position of AE classes dots: AE classes further away from origin (0, 0) in a given direction indicates a high contribution to explain the variability in that dimension;
2. Identify level of similarity among toxicity profiles of treatments based on how close their toxicity profiles are from the origin (0, 0), which represents a hypothetical average toxicity profile;
3. Compare toxicity profiles of treatments based on their position on each dimension.

Notice that distances between treatment profiles and AE classes are not meaningful because they are different spaces. Figures S1 and S2 based on toy data illustrate these steps.

### R-package

We developed the R-package *visae*, an acronym for visualizing AE, aiming to provide statistical software to quickly deploy Shiny applications making our visual approach interactively available for AE reporting. Currently, there are two R-packages specific for CA: *ca* [31] and *FactoMineR* [32]. The R-package *visae* is built based on *ca*. Although both R-packages ca and *FactoMineR* provides CA biplots, the R-package visae makes available the pre-processing of AE data to construct tables such as Table 1 and interactive Shiny application to explore CA configurations.

Interactive applications allow statisticians and non-statisticians to easily collaborate to investigate several configurations for CA, and select the ones that are more informative to them. Therefore, the R-package *visae* provides a general framework for CA allowing statisticians easily interact with their collaborators.

The function run_ca has seven arguments, with four of them required to execute the Shiny application:

- *data*: a data.frame or tibble object in a long format;
- *group*: unquoted variable name in the data that corresponds to the group variable;
- *id:* unquoted variable name in the data that corresponds to the patient identification variable;
- *ae grade*: unquoted variable name in the data that corresponds to AE grade class;

While the other three inputs can be used in any combination,

- *ae domain:* unquoted variable name in the data that corresponds to AE domain class;
- *ae term*: unquoted variable name in the data that corresponds to AE term class;
- *ae cycle*: unquoted variable name in the data that corresponds to AE cycle.

For example, an R user can open the Shiny application as below:

- library(visae)
- library (magrittr)

- patient_id <- 1:100
- group <- c (rep(“A”, 50), rep(“B”, 50))
- grade <- sample(1:5, size = 100, replace = TRUE)
- domain <- sample(c(“C”, “D”), size = 100,replace = TRUE)
- term <- sample(c(“E”, “F”, “G”, “H”),size = 100, replace = TRUE)

- data <- data.frame (patient_id, group,grade, domain, term)
- head (data, n = 6)

**Table:** 

patient_id	group	grade	domain	term
1	A	3	C	G
2	A	3	C	E
3	A	2	D	G
4	A	2	D	E
5	A	5	D	F
6	A	3	C	H

- data %>% run_ca(group = trt,
  - id = patient_id,
  - ae_grade = grade,
  - ae_domain = domain,
  - ae_term = term)

All the contribution biplots and relative frequency tables presented in the next sections were generated using our Shiny application.

### Data sets

Data from two randomized clinical trials from the National Surgical Adjuvant Breast and Bowel Project (NSABP) were used as case examples for this analysis:

### R04

NSABP R04 was a Phase III randomized 2x2 factorial trial comparing neoadjuvant radiation therapy (RT) in combination with either Cape or 5-FU with or without Oxa in patients with rectal cancer (NCT00058474) [19]. AE data (CTCAE version 4.0) were collected at a single time point and included a list of 50 AEs of special interest that were selected a priori and evaluated after chemoradiation treatment within 2 weeks of surgery. Additional details of the trial are reported elsewhere [19].

### B35

NSABP B35 was a Phase III double-blind, randomized, placebo-controlled trial comparing daily oral tamoxifen with oral anastrozole for 5 years in postmenopausal women with hormone receptor-positive ductal carcinoma in situ treated with lumpectomy and radiation therapy (NCT00053898) [33]. Adverse events were assessed every 6 months during therapy and 6 months after the last dose of therapy using a list of predefined AEs (e.g., depression, thromboembolic events, GI disturbance, hot flashes, joint pain, vaginal dryness), graded per Common Toxicity Criteria (CTC) v2.0. Non-adherent patients were defined as patients that stopped treatment early before 5 years for reasons other than disease progression or death.

## Results

We illustrate the main concepts of correspondence analysis when used to represent AE data comparing treatments in the R04 trial and discuss the interaction between treatment and adherence using data from the B35 trial. Analyses at five levels of refinement are presented, but only contingency tables for AE classes defined based on grades are shown. For all other AE classes, contingency tables are presented as Additional file.

### R04

We performed CA considering the different AE class definitions discussed previously with four treatments: 5-FU, Cape, 5-FU+Oxa and Cape+Oxa. Table 2 shows the total number of AE classes for each definition with the percentage of explained inertia for each of the three dimensions. When we define AE classes solely based on AE grades, Table 1 will have 10 (2 *× I* ) rows with 5 AE grades (each one adds its complementary), such that a two-dimensional display describes 98.23% of the variability among treatments with a 1.77% loss of information when dimension 3 of the toxicity space is ignored. The loss of information when representing the toxicity space in a two-dimensional display increases as the level of complexity for AE classes increases. For all AE class definitions, the loss of information is no more than 21% making the two-dimensional display an acceptable representations of the toxicity space.

**Table 2:** AE classes and their decomposition of total inertia into 3 dimensions for R04 trial

AE Class	# AE classes	Dim 1	Dim 2	Dim 3
Grade	5	87.77	10.46	1.77
Domain	21	84.65	10.97	4.38
Domain + Grade	61	66.47	22.94	10.59
Term	209	52.29	28.03	19.68
Term + Grade	313	48.92	30.26	20.82

**Fig. 1:**
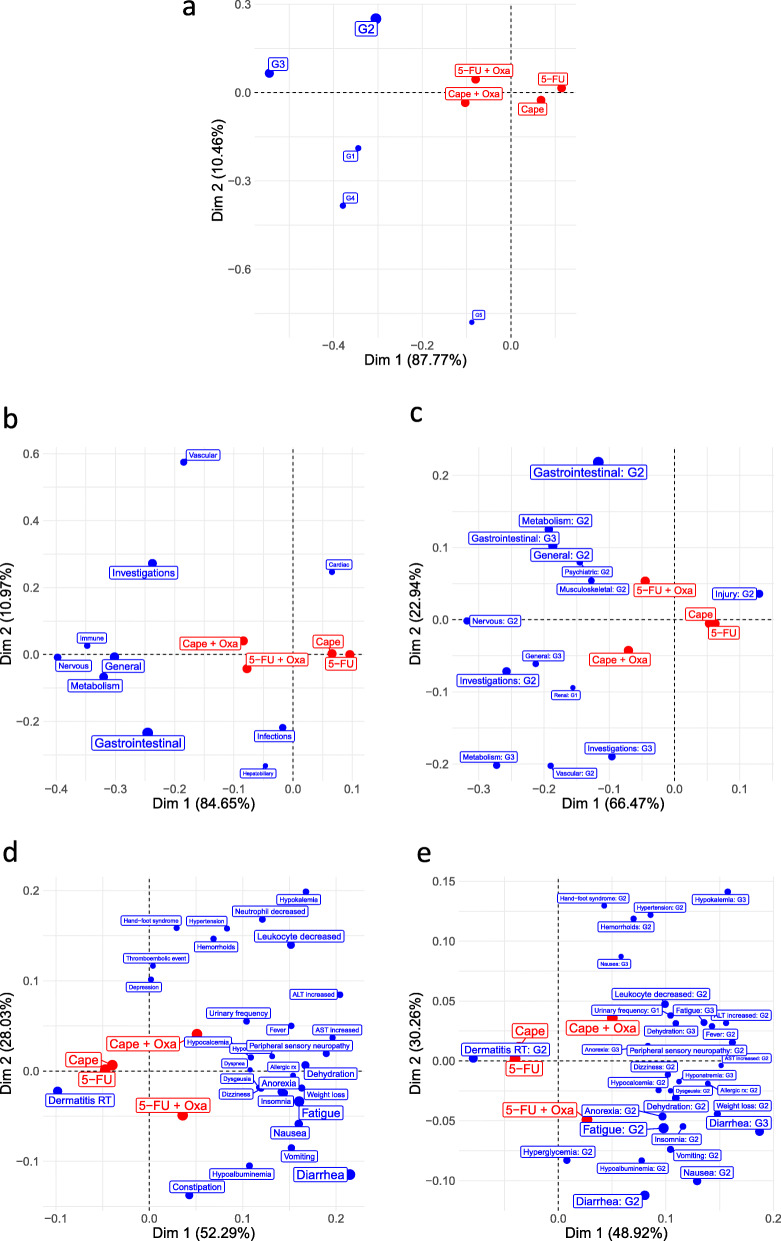
Asymmetric contribution biplots for AE data from R04 trial – **a** AE class defined by AE grades; **b** AE class defined by AE domains with contribution at least 4.76%; **c** AE class defined by AE domains and grades with contribution and relative frequency at least 3.22%; **d** AE class defined by AE terms with contribution and relative frequency at least 0.96%; **e** AE class defined by AE terms and grades with contribution at least 0.64% and relative frequency at least 0.96%

Initially, we assume AE classes based on AE grades as showed in Table 3. In Fig. 1a, main differences are observed in dimension 1 such that discrepancies among treatments are small because their treatment profiles are posed close to each other and they are near to the origin, which represents the average treatment. In dimension 1, ignoring grade 1 AEs that were under-reported, treatments can be ordered based on their relative positions indicating that 5-FU is associated with the lowest frequencies for all AE grades, while Cape+Oxa and 5-FU+Oxa present higher frequency of grade 2, 3, 4 and 5 AEs than their corresponding single agents. Moreover, Cape is associated with higher frequency of grade 5 AEs than 5-FU, Cape+Oxa is associated with higher frequency of grades 1, 4 and 5 AEs, and 5-FU+Oxa with higher frequency of grade 2 AEs than other treatments.

**Table 3:** Percentage (%) of AE grades by treatment in R04 trial

AE Class	Treatment				Average
	5-FU	5-FU + Oxa	Cape	Cape + Oxa	
G1	1.22	2.75	1.23	3.96	2.29
G2	60.67	74.01	63.08	70.73	67.12
G3	25.31	38.53	27.39	39.94	32.79
G4	0.61	3.06	2.15	4.27	2.52
G5	0.31	0.31	1.23	1.52	0.84

While Table 3 is small enough to be understood without a CA biplot, we are interested in more refined AE classes. We define AE classes based on domains as showed in Fig. 1b, with main differences among treatments in dimension 1. Single agents 5-FU and Cape are not very different between them, but both of them differ from their combinations with Oxa. Treatment combinations are associated with AEs in the domains Immune, Nervous, General, Metabolism, Gastrointestinal and Investigations; such that Cape+Oxa and 5-Fu+Oxa are associated in a larger extent with AEs in the domain Investigations and Gastrointestinal, respectively. In addition, Cape+Oxa is associated with the domain Vascular and 5-FU+Oxa is associated with domains Infections and Hepatobiliary.

In Fig. 1c, AE classes are defined based on the combination between domains and grades, with differences in both dimensions. Similar interpretation as Fig. 1b can be outlined, except that the domains Metabolism and General are posed into different quadrants when broken down by grades: Metabolism:G2 and General:G2 are associated with 5-FU+Oxa, while Metabolism:G3 and General:G3 with Cape+Oxa. Moreover, it is possible to observe clustering of domains: (i) domains in the top left quadrant have higher frequency among patients that received 5-FU+Oxa; (ii) domains in the bottom left quadrant have higher frequency for Cape+Oxa; (iii) domain Nervous:G2 is associated with both treatments, and Injury:G2 with their single agents counterparts.

Next, we show AE terms in Fig. 1d with differences in both dimensions. As in the previous analyses, differences between Cape and 5-FU are small such that both are associated with higher frequency of Dermatitis RT when compared to their combinations with Oxa. Treatment combinations are both associated with several of AE terms including Peripheral sensory neuropathy, Diarrhea, Dehydration, Nausea, Vomiting, and Fatigue; such that 5-Fu+Oxa is associated in a larger extent with Diarrhea, Vomiting and Nausea. Cape+Oxa is also associated with Hand-foot syndrome. In particular, anal pain and abdominal pain were not identified in CA configurations that were not overpopulated by AE terms, indicating that their contribution to treatment differences is low in dimension 1 and 2 even though they have high average frequency of 19.42% and 7.49%, respectively. The highest contribution of anal pain is 1.22% in the third dimension.

Finally, we broke down the AE terms by adding their grades. In Fig. 1e, AE classes are defined based on terms and grades. Differences among treatments are found in both dimensions. When comparing Fig. 1e to d, we highlight the term Fatigue that was divided into Fatigue:G2 and G3 associated with 5-FU+Oxa and Cape+Oxa, respectively; the term Nausea was also divided into Nausea:G2 and G3 associated with 5-FU+Oxa and Cape+Oxa, respectively. Furthermore, the AE term Diarrhea is divided in Diarrhea:G3 associated with both 5-FU+Oxa dn Cape+Oxa, and Diarrhea:G2 associated with 5-Fu+Oxa. Furthermore, the distance between 5-FU and Cape is smaller than the distance between 5-FU+Oxa and Cape+Oxa in all CA configurations, which can be interpreted as an interaction effect of Oxa.

### B-35

We performed CA considering the different AE class definitions discussed in the previous section comparing four groups: adherent Anastrozole and Tamoxifen, and their non-adherent counterparts based on AEs reported at cycle 1. Table 4 shows the total number of AE classes for each definition with the percentage of explained inertia for each of the three dimensions. For all AE class definitions, the loss of information is at most 11% for the highest complexity level of the toxicity space.

**Table 4:** AE classes and their decomposition of total inertia into 3 dimensions for B35 trial

AE Class	# AE classes	Dim 1	Dim 2	Dim 3
Grade	3	92.57	6.98	0.45
Domain	20	62.35	34.46	3.19
Domain + Grade	60	55.73	39.80	4.47
Term	214	47.20	43.01	9.79
Term + Grade	384	48.18	41.52	10.30

**Fig. 2:**
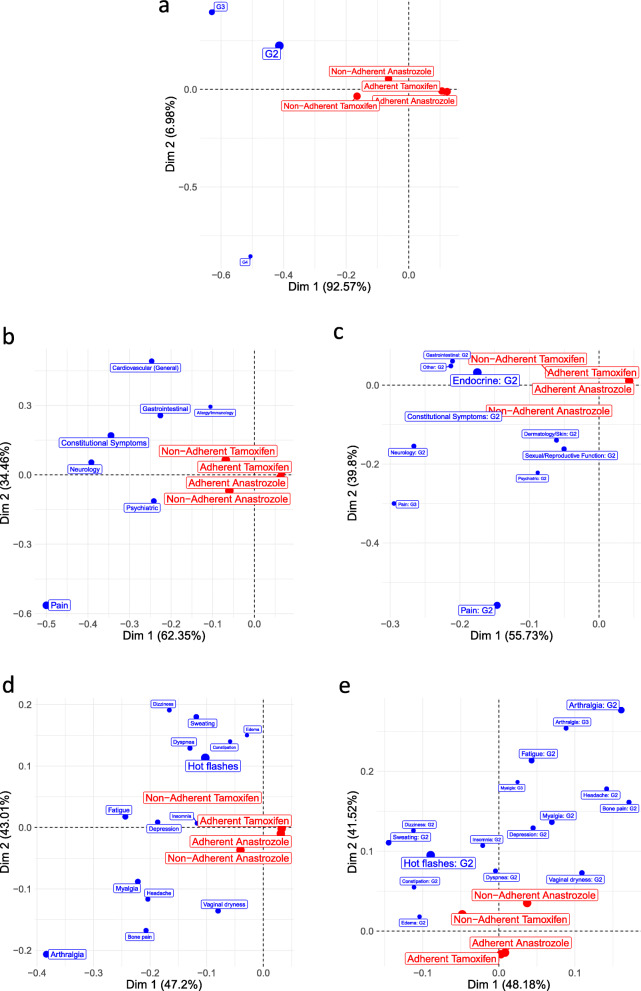
Asymmetric contribution biplots for AE at cycle 1 from B35 trial – **a** AE class defined by AE grades; **b** AE class defined by AE domains with contribution at least 5.0%; **c** AE class defined by AE domains and grades with contribution and relative frequency at least 1.64%; **d** AE class defined by AE terms with contribution at least 0.94% and relative frequency at least 0.47%; **e** AE class defined by AE terms and grades with contribution and relative frequency at least 0.52%

As previously, we assume AE classes based on AE grades as showed in Table 5. Because of small size of contingency table, the first dimension is enough to understand the differences among treatments in Fig. 2a. Adherent Tamoxifen and Anastrozole groups are quite similar to each other and are close to the average treatment. Both non-adherent groups are on the left quadrants indicating higher frequency of AE grades 2, 3 and 4. Nonetheless, non-adherent Tamoxifen and Anastrozole are in different quadrants showing that their discrepancies from the average treatment are grade specific: non-adherent Tamoxifen is more associated with grade 4 AEs, while non-adherent Anastrozole is more associated with grade 2 and 3 AEs. Table 5 leads to similar conclusions.

**Table 5:** Percentage (%) of AE grades at cycle 1 by treatment in B35 trial

AE class	Adherent		Non-adherent		Average
	Anastrozole	Tamoxifen	Anastrozole	Tamoxifen	
G2	38.22	40.69	49.66	52.23	45.20
G3	2.22	3.91	10.61	11.94	7.63
G4	0.188	0.279	0.903	3.279	1.162

In Fig. 2b, AE classes are defined based on domains, such that differences between adherent and non-adherent groups are showed in the first dimension and differences between non-adherent Anastrozole and non-adherent Tamoxifen in the second dimension. Non-adherent groups are associated with higher frequency of AEs in the domains Pain, Neurology, Constitutional Symptoms, Psychiatric, Cardiovascular, Gastrointestinal, Allergy/Immunology. In particular, non-adherent Anastrozole is more associated with the domains Psychiatric and Pain, while non-adherent Tamoxifen is associated with the domains Gastrointestinal, Allergy/Immunology and Cardiovascular.

Figure 2c combines AE grades to the AE domains as AE classes. Differences are found in both dimensions with similar interpretation from Fig. 2b. In particular, we highlighted the domain Pain from Fig. 2b that was broken down into Pain:G3 associated with both non-Aaherent Tamoxifen and non-adherent Anastrozole, while Pain:G2 is associated only with non-adherent Anastrozole..

In Fig. 2d, AE classes are defined based on terms. Differences are found in both dimensions with similar interpretation from Fig. 2b. The AE terms can be divided into three clusters: (i) Dizziness, Sweating, Edema, Constipation, Dyspnea, Hot flashes and Radiation dermatitis associated with non-adherent Tamoxifen; (ii) Arthralgia, Bone Pain, Headache, Myalgia, Varginal dryness are associated with non-adherent Anastrozole; (iii) Fatigue, Depression, and Insomnia are associated with both non-adherent groups.

As the last step, Fig. 2e combines terms and grades. There is a change in the interpretation of the dimensions: discrepancies between non-adherent Anastrozole and Tamoxifen becomes more relevant to explain the variability among groups than the differences between adherent and their non-adherent counterparts, which yields the reverse interpretation of the dimensions from Fig. 2a-d. Furthermore, clusters of AEs associated with non-adherent Anastrozole and Tamoxifen are observed similar to Fig. 2d.

## Discussion

We proposed stacked CA using contribution biplots as a tool to explore differences in AE data among treatments in clinical trials. We defined five levels of refinement for the analysis based on AE grades, domains, terms and their combinations. In addition, we developed a Shiny application built in an R-package to interactively investigate CA configurations based on the contribution to the explained variance and relative frequency of AEs, and we made it publicly available on CRAN. Phillips et al [2] have found that only 12% among 184 clinical trials published in major medical journals between 2015 and 2016 showed graphical presentations. We expect to improve AE reporting through statistical graphical displays and easy-to-use software that can also be transformed into web applications as suggested by the Consolidated Standards of Reporting Trials (CONSORT) Harm extension [6]. Furthermore, the proposed analysis could also be applied to patient reported outcomes (PRO) such as PRO-CTCAE [34].

Morever, we illustrated the use of stacked CA for different goals in the R04 and B35 clinical trials. In our examples, toxicity spaces representing contingency tables at the highest level of refinement of AE classes - term and grade combination - have three dimensions such that the loss of information when using CA biplots was 20.82% in R04 and 10.30% in B35, which are within the threshold of 30% as proposed by some authors. In R04 trial, CA biplots displayed the differences in AE patterns between single agent treatments and their combinations with Oxa at all levels of AE classes, such that an interaction effect when adding Oxa to Cape/5-FU was identified. In the B35 trial, CA biplots showed the discrepancies between non-adherent and adherent Tamoxifen and Anastrozole. Different patterns for non-adherent Anastrozole and Tamoxifen were observed contrasting with their adherent counterparts. Interestingly, CA biplots identified expected differences in AE frequency between treatments (e.g., Arthralgia associating with non-adherent Anastrozole and Hot Flashes associated with non-adherent Tamoxifen), but also others that were less expected (e.g., Depression and fatigue associated with Anastrozole and Dizziness with Tamoxifen).

The main goal of CA in the context of AEs is to visualize associations between treatments and AEs while controlling the loss of information due the dimension reduction of the toxicity space. The loss of information is an increasing function of the level of refinement of AE classes. The several levels of AE classes could give researchers insights regarding the discrepancies among treatments. While CA with AE grades presents a more understandable biplot, it does not discriminate the toxicity profiles enough. On the other hand, CA biplots with AE terms and grades presents a lot of information that might make harder to draw conclusions at the same time allowing one to understand detailed differences among groups. A possible compromise is CA biplots with domains or domains and grades that indicate enough differences among treatment without being visually overwhelming.

Ideally, CA biplots with AE classes defined based on AE grades and their combinations with terms/ domains would show AEs following the grade ordering. Nonetheless, such pattern is rarely observed even in CA biplots that are based solely on AE grades with large variability explained by dimension 1. The lack of ordering is expected, although not intuitive, because associations, which are shown by CA biplots, between treatments and AE classes defined based on AE grades are often not ordered. At first glance, the understanding of CA biplots might be misleading, but we believe that annotated toy CA biplots as presented in this work will help researchers to interpret results.

We have not discussed inference based on CA given its limited scope. Few authors have [35–37] presented inferential procedures using bootstrap to provide confidence regions on CA biplots with poor performance when dealing with sparse matrices. Also, we did not study the association pattern within AE classes for a treatment and multiple AE of the same grade are not taken into account if a CA biplot is based on more than one treatment cycle. In future work, we plan to apply joint CA to visualize association pattern within AE classes by treatments and analysis of matched matrices to compare such patterns between treatments, respectively.

## Conclusion

CA with contribution biplot is an effective tool that can be used to summarize AE data in a two-dimensional display while minimizing the loss of information and interpretation. It is general enough to be applied to a variety of drugs classes and diseases. Instead of lengthy frequency tables presented as supplemental material of trial reports, CA biplots for AE classes defined based on either terms or the combination between terms and grades could be presented, so the data can be examined visually. Furthermore, CA could be used to help investigators to select AE classes to be summarized based on objective criteria given by their contributions to the explained variance and relative frequencies. In this way, AE reporting would be more consistent across studies. A drawback of such strategy is that it could miss AE classes with high frequencies, but low contribution such as Anal and Abdominal Pain in R04 trial. Therefore, clinical input such as relatedness to treatment and severity of AEs should also be considered and several configurations of CA biplots should be investigated such that conclusions need to be double-checked with frequencies tables, which are also provided in the Shiny app in the R-package *visae*.

## Supplementary Information

**Additional file 1.**

## Data Availability

The data that support the findings of this study are available from NRG Oncology but restrictions apply to the availability of these data, which were used under license for the current study, and so are not publicly available. Requests for the data, however, can be made to NRG Oncology at https://www.nrgoncology.org/Resources/Ancillary-Projects-Data-Sharing-Application.

## Abbreviations

AE: Adverse Event
CA: Correspondence Analysis
CTCAE: Common Terminology Criteria for Adverse Events
CRAN: Comprehensive R Archive Network
RCT: Randomized Clinical Trial
Cape: Capecitabine
5-FU: 5-fluorouracil
Oxa: Oxaliplatin
RT: Radiation therapy
CTC: Common Toxicity Criteria

## References

[1] of Health, U.D., Services, H (2009). National Cancer Institute: Common Terminology Criteria for Adverse Events (CTCAE) Version 4.0.

[2] Phillips R, Hazell L, Sauzet O, Cornelius V (2019). Analysis and reporting of adverse events in randomised controlled trials: a review. BMJ Open.

[3] Lee S, Hershman D, Martin P, Leonard J, Cheung Y (2011). Toxicity burden score: a novel approach to summarize multiple toxic effects. Ann Oncol.

[4] Thanarajasingam G, Atherton PJ, Novotny PJ, Loprinzi CL, Sloan JA, Grothey A (2016). Longitudinal adverse event assessment in oncology clinical trials: the toxicity over time (toxt) analysis of alliance trials ncctg n9741 and 979254. Lancet Oncol.

[5] Gresham G, Diniz MA, Razaee ZS, Luu M, Kim S, Hays RD, Piantadosi S, Tighiouart M, Yothers G, Ganz PA (2020). Evaluating treatment tolerability in cancer clinical trials using the toxicity index. J Natl Cancer Instit.

[6] Ioannidis JP, Evans SJ, Gøtzsche PC, O’neill RT, Altman DG, Schulz K, Moher D (2004). Better reporting of harms in randomized trials: an extension of the consort statement. Ann Intern Med.

[7] Amit O, Heiberger RM, Lane PW (2008). Graphical approaches to the analysis of safety data from clinical trials. Pharm Stat.

[8] Zink RC, Wolfinger RD, Mann G (2013). Summarizing the incidence of adverse events using volcano plots and time intervals. Clin Trials.

[9] Karpefors M, Weatherall J (2018). The tendril plot—a novel visual summary of the incidence, significance and temporal aspects of adverse events in clinical trials. J Am Med Inform Assoc.

[10] Greenacre M (1992). Correspondence analysis in medical research. Stat Methods Med Res.

[11] Sourial N, Wolfson C, Zhu B, Quail J, Fletcher J, Karunananthan S, Bandeen-Roche K, Beland F, Bergman H (2010). Correspondence analysis is a useful tool to uncover the relationships among categorical variables. J Clin Epidemiol.

[12] Hirsch O, Bosner S, Hüllermeier E, Senge R, Dembczynski K, Donner-Banzhoff N (2011). Multivariate modeling to identify patterns in clinical data: the example of chest pain. BMC Med Res Methodol.

[13] Befus M, Mukherjee D, Herzig C, Lowy F, Larson E (2017). Correspondence analysis to evaluate the transmission of staphylococcus aureus strains in two New York state maximum-security prisons. Epidemiol Infect.

[14] Fellenberg K, Hauser NC, Brors B, Neutzner A, Hoheisel JD, Vingron M (2001). Correspondence analysis applied to microarray data. Proc Natl Acad Sci.

[15] Busold CH, Winter S, Hauser N, Bauer A, Dippon J, Hoheisel JD, Fellenberg K (2005). Integration of go annotations in correspondence analysis: facilitating the interpretation of microarray data. Bioinformatics.

[16] Horita T, Gaballah MH, Fukuta M, Kanno S, Kato H, Takamiya M, Aoki Y (2020). Time course analysis of large-scale gene expression in incised muscle using correspondence analysis. PLoS One.

[17] Le-Rademacher JG, Hillman S, Storrick E, Mahoney MR, Thall PF, Jatoi A, Mandrekar SJ (2020). Adverse event burden score—a versatile summary measure for cancer clinical trials. Cancers.

[18] Russell MM, Ganz PA, Lopa S, Yothers G, Ko CY, Arora A, Atkins JN, Bahary N, Soori G, Robertson JM (2015). Comparative effectiveness of sphincter-sparing surgery versus abdominoperineal resection in rectal cancer: patient-reported outcomes in national surgical adjuvant breast and bowel project randomized trial r-04. Ann Surg.

[19] Allegra CJ, Yothers G, O’Connell MJ, Beart RW, Wozniak TF, Pitot HC, Shields AF, Landry JC, Ryan DP, Arora A (2015). Neoadjuvant 5-fu or capecitabine plus radiation with or without oxaliplatin in rectal cancer patients: a phase iii randomized clinical trial. J Natl Cancer Inst.

[20] Cella D, Land SR, Chang C-H, Day R, Costantino JP, Wolmark N, Ganz PA (2008). Symptom measurement in the breast cancer prevention trial (bcpt)(p-1): psychometric properties of a new measure of symptoms for midlife women. Breast Cancer Res Treat.

[21] Land SR, Cronin WM, Wickerham DL, Costantino JP, Christian NJ, Klein WM, Ganz PA (2011). Cigarette smoking, obesity, physical activity, and alcohol use as predictors of chemoprevention adherence in the national surgical adjuvant breast and bowel project p-1 breast cancer prevention trial. Cancer Prev Res.

[22] Margolese RG, Cecchini RS, Julian TB, Ganz PA, Costantino JP, Vallow LA, Albain KS, Whitworth PW, Cianfrocca ME, Brufsky AM, Gross HM, Soori GS, Hopkins JO, Fehrenbacher L, Sturtz K, Wozniak TF, Seay TE, Mamounas EP, Wolmark N (2016). Anastrozole versus tamoxifen in postmenopausal women with ductal carcinoma in situ undergoing lumpectomy plus radiotherapy (nsabp b-35): a randomised, double-blind, phase 3 clinical trial. Lancet.

[23] Ganz PA, Cecchini RS, Julian TB, Margolese RG, Costantino JP, Vallow LA, Albain KS, Whitworth PW, Cianfrocca ME, Brufsky AM, Gross HM, Soori GS, Hopkins JO, Fehrenbacher L, Sturtz K, Wozniak TF, Seay TE, Mamounas EP, Wolmark N (2016). Patient-reported outcomes with anastrozole versus tamoxifen for postmenopausal patients with ductal carcinoma in situ treated with lumpectomy plus radiotherapy (nsabp b-35): a randomised, double-blind, phase 3 clinical trial. Lancet.

[24] Land SR, Walcott FL, Liu Q, Wickerham DL, Costantino JP, Ganz PA (2016). Symptoms and qol as predictors of chemoprevention adherence in nrg oncology/nsabp trial p-1. J Natl Cancer Inst.

[25] Greenacre M (2013). Contribution biplots. J Comput Graph Stat.

[26] Hirschfeld HO. A connection between correlation and contingency. In: Mathematical Proceedings of the Cambridge Philosophical Society. Cambridge University Press; 1935;31:520–4.

[27] Benźecri J-P (1973). L’analyse des Donńees.

[28] Greenacre MJ (1984). Theory and applications of correspondence analysis, 1st edition edn.

[29] Greenacre MJ. Correspondence Analysis in Practice. 1st ed: CRC press; 1993.

[30] Greenacre MJ (1993). Biplots in correspondence analysis. J Appl Stat.

[31] Nenadic O, Greenacre M (2007). Correspondence analysis in r, with two- and three-dimensional graphics: the ca package. J Stat Softw.

[32] Le S, Josse J, Husson F (2008). FactoMineR: A package for multivariate analysis. J Stat Software.

[33] Forbes JF, Sestak I, Howell A, Bonanni B, Bundred N, Levy C, Von Minckwitz G, Eiermann W, Neven P, Stierer M (2016). Anastrozole versus tamoxifen for the prevention of locoregional and contralateral breast cancer in postmenopausal women with locally excised ductal carcinoma in situ (ibis-ii dcis): a double-blind, randomised controlled trial. Lancet.

[34] Dueck AC, Mendoza TR, Mitchell SA, Reeve BB, Castro KM, Rogak LJ, Atkinson TM, Bennett AV, Denicoff AM, O’Mara AM (2015). Validity and reliability of the us national cancer institute’s patient-reported outcomes version of the common terminology criteria for adverse events (pro-ctcae). JAMA Oncol.

[35] Ringrose TJ (2012). Bootstrap confidence regions for correspondence analysis. J Stat Comput Simul.

[36] Lombardo R, Ringrose T (2012). Bootstrap confidence regions in non-symmetrical correspondence analysis. Electron J Appl Stat Analysis.

[37] Beh EJ, Lombardo R (2015). Confidence regions and approximate p-values for classical and non symmetric correspondence analysis. Commun Stati Theory Methods.

