# Supplementary material for: Visualizing adverse events in clinical trials using correspondence analysis with R-package visae
Source: BMC Med Res Methodol. 2021 Nov 9;21:244. doi: 10.1186/s12874-021-01368-w (PMC8579548; doi:10.1186/s12874-021-01368-w)
Supplement: Supplementary file 1 — Additional file 1. [file 12874_2021_1368_MOESM1_ESM.pdf]

RESEARCH

Visualizing adverse events in clinical trials using correspondence analysis with R-package visae - Additional file

Márcio A. Diniz[??*], Gillian Gresham[??], Sungjin Kim[??], Michael Luu[??], N. Lynn Henry[??], Mourad Tighiouart[??], Greg Yothers[??], Patricia A. Ganz[??] and André Rogatko[??]

[*]Correspondence: marcio.diniz@cshs.org
[??]Samuel Oschin Comprehensive Cancer Center, Cedars-Sinai Medical Center, Los Angeles, CA, US
Full list of author information is available at the end of the article

Mathematical Description

The goal of CA is to graphically represent contingency tables. In particular, for AE data, we consider a contingency table of I AE classes and J treatments as shown in Table S1. For now, AE class will be a generic label to indicate a variable that classifies AE into categories following a given criteria.

AE class	Treatment (T) 1	...	J	Total per AE
1	n_{11}	...	n_{1J}	$n_{1.}$
⋮	⋮	⋮	⋮	⋮
I	n_{I1}	...	n_{IJ}	$n_{I.}$
Total per treatment	$n_{.1}$	...	$n_{.J}$	$n_{..}$

Table S1: Contingency table $I \times J$ with row and column marginals

The next step would be to the define treatment profiles as relative frequencies, $p_{ij} = n_{ij}/n_{.j}$ for $i = 1, \ldots, I$ and $j = 1, \ldots, J$. However, the column marginals in Table S1 are the total number of AE instead of the total number of patients, and investigators are interested in contingency tables with the patient as the sample unit. Therefore, we need to redefine the contingency table before proceeding to apply correspondence analysis. First, we calculate a relative frequency table for each AE class i given the total number of patients in each treatment as defined in Table S2.

AE class	Treatment (T) 1	...	J	Total per AE
i	π_{i1}	...	π_{ij}	$\sum_{j=1}^{J} \pi_{ij}$
i^C	$1 - \pi_{i1}$	...	$1 - \pi_{iJ}$	$J - \sum_{j=1}^{J} \pi_{ij}$
Total per treatment	1	...	1	J

Table S2: Contingency table $2 \times J$ with row and column marginals

where $\pi_{ij} = \sum_{l=1}^{N_j} I_{(n_{ijl}>0)}/N_j$ is the relative frequency of AE_i class for treatment T_j, n_{ijl} is the frequency of AE class i for patient l receiving treatment T_j, and N_j is the total number of patients receiving treatment T_j for $i = 1, \ldots, I$ and $j = 1, \ldots, J$. Then, AE class specific contingency tables such as Table S2 can be stacked generating an extended table for all AE classes.

Following Greenacre [?], we will apply CA on stacked tables such as Table S3.

AE class	Treatment (T) 1	...	J	Total per AE
1	π_{11}	...	π_{1J}	$\sum_{j=1}^{J} \pi_{1j}$
1^c	$1-\pi_{11}$	...	$1-\pi_{1J}$	$J-\sum_{j=1}^{J} \pi_{1j}$
⋮	⋮	⋮	⋮	⋮
I	π_{I1}	...	π_{IJ}	$\sum_{j=1}^{J} \pi_{Ij}$
I^c	$1-\pi_{I1}$	...	$1-\pi_{IJ}$	$J-\sum_{j=1}^{J} \pi_{Ij}$
Total per treatment	I	...	I	IJ

Table S3: Contingency table $2I \times J$ with row and column marginals

We define column profiles as relative frequencies for each treatment,

$$\mathbf{p}_{.\mathbf{j}} = \begin{bmatrix} p_{ij} \\ p_{i^c j} \end{bmatrix}_{i=1}^{I} = \begin{bmatrix} \pi_{ij}/I \\ \pi_{i^c j}/I \end{bmatrix}_{i=1}^{I} = \begin{bmatrix} \pi_{ij}/I \\ (1-\pi_{ij})/I \end{bmatrix}_{i=1}^{I} \tag{1}$$

resulting in a $2I \times J$ correspondence matrix $\mathbf{P} = [p_{.j}]_{j=1}^{J}$ associated with Table S3. Although CA can be based either on column or row profiles, we will focus only on the analysis for column profiles. Both analyses are mathematically equivalent, but they lead us to different interpretations. The interpretation of Table S3 is asymmetric: we are interested in studying the differences in treatment profiles that lie in the space generated by the column profiles, which will be denoted as toxicity space.

The toxicity space has dimension $K = min\{I, J\}$, such that the vertices of such space are given by extreme treatment profiles, i.e., treatment profiles that have one as a relative frequency for AE class i and zero for all other AE classes for $i = 1, \ldots, I$. The toxicity space can be understood using summary measures of location and dispersion as follows. First, we calculate column marginal relative frequencies, denoted as masses in CA,

$$\mathbf{c}_{J\times 1} = [c_j]_{j=1}^{J} = \left[\frac{I}{IJ}\right]_{j=1}^{J} = \left[\frac{1}{J}\right]_{j=1}^{J} \tag{2}$$

The masses associated with each treatment are the same, independent of the number of patients or number of AEs, which is desirable because there is no particular reason to give a higher weight for a specific group in our analysis. Although treatments are often equally randomized in clinical trials, we also can define groups as treatment adherent or non-adherent which will not have the same number of patients as will be discussed in the next section.

Then, the average toxicity profile is defined as the row marginal relative frequencies. It also can be calculated as a weighted average of treatment profiles (1) with weights given by the treatment masses (2),

$$\begin{aligned} \mathbf{r}_{2I\times 1} &= \begin{bmatrix} r_i \\ r_{i^c} \end{bmatrix}_{i=1}^{I} = \sum_{j=1}^{J} c_j p_{.j} \\ &= \begin{bmatrix} \bar{\pi}_{i.}/I \\ (1-\bar{\pi}_{i.})/I \end{bmatrix}_{i=1}^{I}, \end{aligned} \tag{3}$$

where $\bar{\pi}_{i.} = \frac{1}{J}\sum_{j=1}^{J}\pi_{ij}$.

In CA, the distance of treatment profiles from the average profile is based on a weighted Euclidean distance known as the χ^2-distance:

$$\chi^2\text{-distance}(\mathbf{p_{.j}}, \mathbf{r}) = \sqrt{\frac{(p_{1j} - r_1)^2}{r_1} + \ldots + \frac{(p_{I^cj} - r_{I^c})^2}{r_{I^c}}}. \quad (4)$$

Then, we can calculate the variability of treatment profiles as a weighted average χ^2 distance (4) of treatment profiles from the average, with weights given by their treatment masses (2). This is known as total inertia in CA and is given by

$$\begin{aligned} \text{total inertia} &= \sum_{j=1}^{J} c_j \chi^2\text{-distance}(p_{.j}, \mathbf{r})^2 \\ &= \frac{1}{IJ}\sum_{j=1}^{J}\sum_{i=1}^{I}\frac{(\pi_{ij} - \bar{\pi}_{i.})^2}{\bar{\pi}_{i.}(1 - \bar{\pi}_{i.})} \end{aligned} \quad (5)$$

which can be interpreted as the average total inertia for all AE classes. Therefore, any toxicity space can be summarized by the average (3) and variance (5) as in any statistical problem. As a next step, we can standardize the correspondence matrix $\mathbf{P}$,

$$\begin{aligned} \mathbf{R} &= \mathbf{D}_c^{-1/2}(\mathbf{P} - \mathbf{c}\mathbf{r}^T)\mathbf{D}_r^{-1/2} \\ &= \mathbf{D}_c^{1/2}(\mathbf{D}_c^{-1}\mathbf{P} - \mathbf{1}\mathbf{r}^T)\mathbf{D}_r^{-1/2} \end{aligned} \quad (6)$$

where $\mathbf{D}_c$ and $\mathbf{D}_r$ are $J \times J$ and $2I \times 2I$ diagonal matrices defined based on (2) and (3), respectively. The standardized residual treatment profiles (6) represent the differences in comparison to the average treatment profile assuming that groups were identical.

Visualizing the standardized residuals treatment profiles in the toxicity space can give us insight regarding the association between treatments and AE classes. Nonetheless, it is not always feasible to display residual treatment profiles when the number of dimensions is greater than three, i.e., four treatment arms ($J \geq 4$) or four AE classes ($I \geq 4$). Moreover, distances between standardized residual treatment profiles are not simple to be evaluated even in a three dimensional space.

In this context, CA seeks the two-dimensional display that minimizes the weighted sum of χ^2-distance (4) between the residual treatment profiles that lie in the K-dimensional toxicity space and their projection on a two-dimensional display candidate, where the weights are given by the treatment masses (2). The solution for this minimization problem is given by biplots [**?**]. The biplot for CA is defined based on the singular value decomposition of (6):

$$\mathbf{R} = \mathbf{U}\mathbf{D}_\alpha\mathbf{V}^T \quad (7)$$

where $\mathbf{U}\mathbf{U}^T = \mathbf{V}^T\mathbf{V} = \mathbf{I}_K$, such that $\mathbf{U}$ and $\mathbf{V}$ are $J \times K$ and $2I \times K$ matrices, respectively, with each column in both matrices representing the dimension k of the

toxicity space for $k = 1, \ldots, K$. Furthermore, $\mathbf{D}_\alpha$ is the diagonal matrix of single values,

$$\mathbf{D}_\alpha = diag(\alpha_1 \geq \ldots \geq \alpha_K) \quad (8)$$

such that $\sum_{k=1}^{K} \alpha_k^2$ is the total inertia given in (5); $\mathbf{D}_c, \mathbf{D}_r$ are diagonal matrices with diagonals given by (2) and (3), respectively. Then, the asymmetric contribution biplot proposed by [**?**] will display two sets of dots: (a) dots representing treatment profiles with coordinates given by $\mathbf{F} = \mathbf{D}_c^{-1/2}\mathbf{U}\mathbf{D}_\alpha$, which are denoted as principal coordinates; and dots representing AE classes with coordinates given by $\mathbf{V}$, such that v_{ik}^2 is the contribution of AE class i in dimension k for $k = 1, \ldots, K$ with $\sum_{i=1}^{I} v_{ik}^2 = \alpha_k^2$. The expected contribution of an AE class is the average contribution assuming that all AE classes have the same contributions, i.e., $1/I$; the expected mass of an class is the average frequency assuming that all AE classes have the same frequency, i.e., $1/J$.

Biplots show the main features of high dimensional data using only two dimensions ($K = 2$) while minimizing the loss of information. The first dimension of the biplot represents the direction with highest inertia (α_1^2) in the toxicity space and the second dimension corresponds to the direction with the second highest inertia (α_2^2). Adding up the inertia of the remaining dimensions ($\sum_{k=3}^{K} \alpha_k^2$) allows us to quantify the loss of information of projecting the toxicity space into a two-dimensional plane. The inertia associated with each dimension (α_k^2) can also be broken down in contributions (v_{ik}^2) of each AE class for $i = 1, \ldots, I$ and $k = 1, \ldots, K$. Therefore, we are able to compare treatment profiles based on the position of their dots relative to the origin, which represents the average treatment (3), and interpret each axis based on the position of dots associated with each AE class.

Finally, AE classes can be defined in Table S3 based on three levels of data aggregation: (a) AE grades, (b) AE domains, (c) AE terms and their combinations. While toxicity profiles can be presented and compared based on tables when AE classes are only defined by AE grades, it is a much more complex task when AE classes are defined by AE domains or AE terms, and their combinations with AE grades. There are 26 domains and 790 AE terms in CTCAE v4, which can generate 130 AE classes when AE domains are combined with AE grades and 3950 AE classes when AE terms are combined with AE grades.

Figures

a

AE class	Treatment				Average treatment
	A	B	C	D	
01	60%	10%	23.30%	0%	23.30%
02	30%	40%	26.70%	10%	26.70%
03	10%	40%	26.70%	30%	26.70%
04	0%	10%	23.30%	60%	23.30%

b

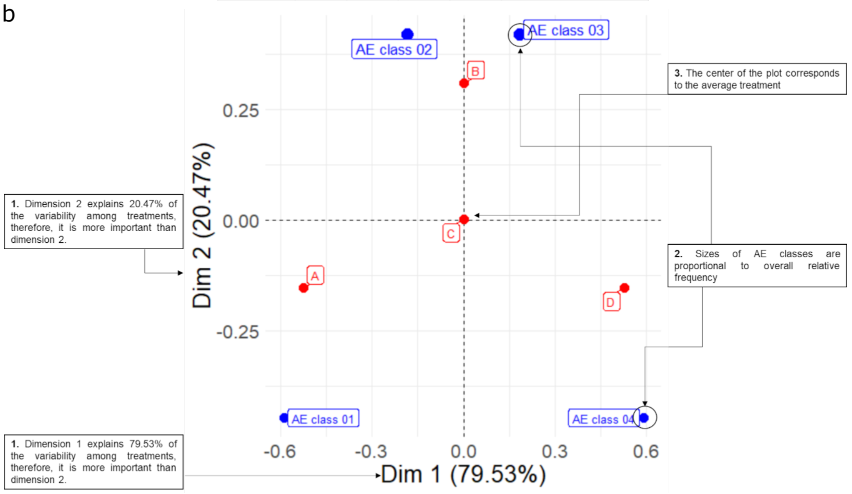

Figure S1: Asymmetric contribution biplot for toy data - a. Contingency table of AE classes by treatment; b. Interpreting variance explained by dimensions, the size of AE classes (blue dots) and the center of CA biplot.

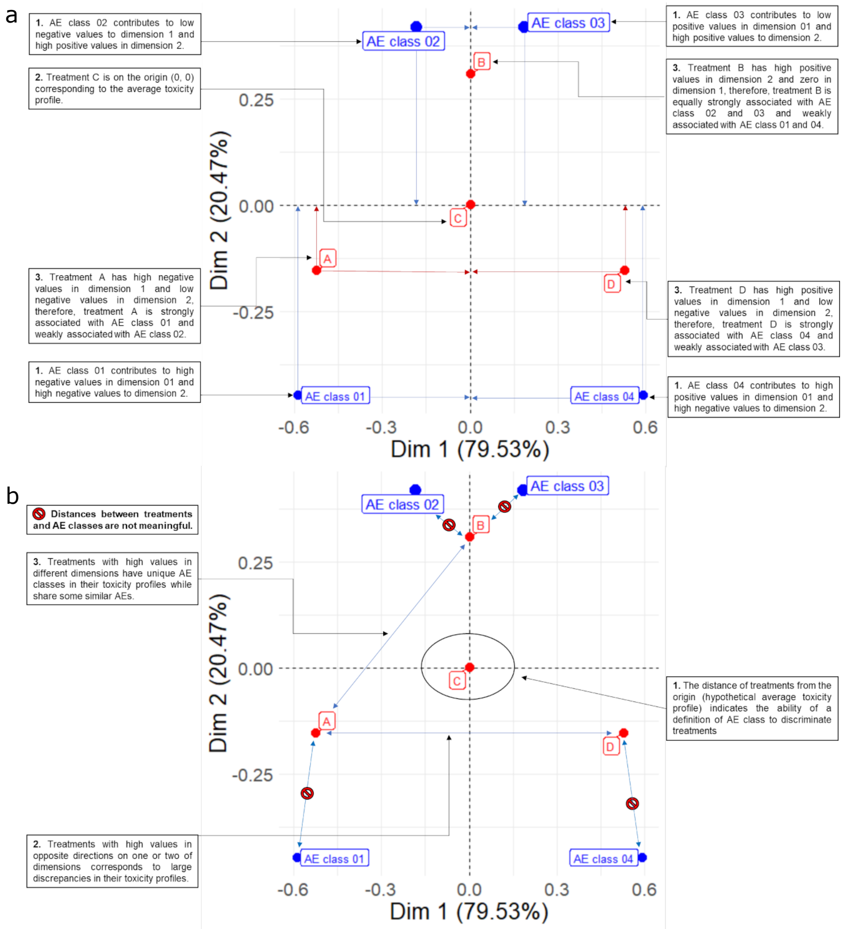

Figure S2: Asymmetric contribution biplot for toy data - a. Interpreting AE classes (blue dots) and groups (red dots) relative to dimension 1 and dimension 2; b. Interpreting the distances among groups (red dots).

Tables

R04

Domain	5-FU (n = 328)	5-FU + Oxa (n = 327)	Cape (n = 325)	Cape + Oxa (n = 328)	Average
Cardiac	0.92	0.31	1.85	1.22	1.07
Gastrointestinal	42.68	60.86	47.08	54.88	51.37
General	19.51	33.33	24.31	33.54	27.67
Hepatobiliary	0.00	0.61	0.31	0.00	0.23
Immune	0.00	3.67	0.31	3.96	1.99
Infections	7.01	8.26	6.46	6.10	6.96
Investigations	19.51	24.46	17.54	29.88	22.85
Metabolism	13.41	24.77	14.46	24.09	19.18
Nervous	3.96	13.15	4.92	13.41	8.86
Vascular	3.35	4.28	4.31	9.45	5.35

Table S4: Percentage (%) of selected AE domains with contribution at least 4.76% by treatment in R04 trial

Domain:Grade	5-FU (n = 328)	5-FU + Oxa (n = 327)	Cape (n = 325)	Cape + Oxa (n = 328)	Average
Gastrointestinal: G2	38.72	52.60	40.92	43.29	43.88
Gastrointestinal: G3	10.37	23.55	14.15	22.26	17.58
General: G2	17.68	28.75	20.92	26.83	23.55
General: G3	1.83	5.20	2.77	7.01	4.20
Injury: G2	14.63	11.31	14.15	9.45	12.39
Investigations: G2	12.50	18.35	10.15	22.56	15.89
Investigations: G3	7.62	7.64	8.31	12.50	9.02
Metabolism: G2	11.59	20.49	12.62	18.29	15.75
Metabolism: G3	3.96	7.34	4.00	12.80	7.03
Musculoskeletal: G2	3.35	6.12	3.38	5.79	4.66
Nervous: G2	3.35	10.70	4.00	12.50	7.64
Psychiatric: G2	1.83	5.81	3.69	5.18	4.13
Renal: G1	1.22	2.45	1.23	3.96	2.21
Vascular: G2	3.35	3.36	0.92	7.01	3.66

Table S5: Percentage (%) of selected AE domain:grade classes with contribution and mass at least 3.22% by treatment in R04 trial

Term	5-FU (n = 328)	5-FU+Oxa (n = 327)	Cape (n = 325)	Cape+Oxa (n = 328)	Average
Alanine aminotransferase increased	0.61	3.06	0.31	5.49	2.37
Allergic reaction	0.00	2.14	0.31	2.44	1.22
Anorexia	4.57	10.40	6.15	10.37	7.87
Aspartate aminotransferase increased	0.00	2.75	0.31	3.96	1.76
Constipation	3.96	9.79	7.69	5.18	6.66
Dehydration	3.96	10.09	5.85	11.28	7.79
Depression	0.92	0.61	2.15	2.13	1.45
Radiation dermatitis	16.46	12.54	15.69	10.67	13.84
Diarrhea	18.60	36.70	20.31	31.40	26.75
Dizziness	0.61	3.06	1.23	3.05	1.99
Dysgeusia	0.00	1.83	0.61	1.83	1.07
Dyspnea	0.61	1.53	0.00	1.83	0.99
Fatigue	15.55	28.44	21.23	27.74	23.24
Fever	1.22	2.75	0.31	4.27	2.14
Hemorrhoids	0.92	0.92	1.85	3.66	1.83
Hypertension	1.22	0.61	0.61	3.35	1.45
Hypoalbuminemia	2.44	5.20	0.92	3.35	2.98
Hypocalcemia	0.92	2.45	0.92	3.05	1.83
Hypokalemia	0.92	1.53	0.92	6.10	2.37
Hyponatremia	0.61	1.83	0.00	2.44	1.22
Insomnia	0.92	4.28	1.54	4.27	2.75
Nausea	5.49	12.84	6.46	11.59	9.10
Neutrophil count decreased	0.61	1.83	2.77	5.79	2.75
Palmar-plantar erythrodysesthesia syndrome	0.30	0.31	2.46	2.74	1.45
Peripheral sensory neuropathy	0.61	5.20	2.15	6.40	3.59
Thromboembolic event	0.30	0.31	2.15	1.83	1.15
Urinary frequency	1.52	2.75	1.54	4.27	2.52
Vomiting	2.74	7.34	1.85	5.79	4.43
Weight loss	2.13	5.81	1.54	6.10	3.90
White blood cell decreased	4.27	5.81	4.00	11.28	6.34

Table S6: Percentage (%) of selected AE terms with contribution and mass at least 0.96% by treatment in R04 trial

Term:Grade	5-FU (n = 328)	5-FU+Oxa (n = 327)	Cape (n = 325)	Cape+Oxa (n = 328)	Average
Alanine aminotransferase increased: G2	0.61	2.14	0.00	3.66	1.60
Allergic reaction: G2	0.00	2.14	0.31	2.44	1.22
Anorexia: G2	3.96	8.87	5.54	7.93	6.57
Anorexia: G3	0.61	1.53	0.61	2.13	1.22
Aspartate aminotransferase increased: G2	0.00	1.83	0.00	2.44	1.07
Dehydration: G2	3.66	7.34	3.69	7.32	5.50
Dehydration: G3	0.30	2.75	2.15	3.96	2.29
Radiation Dermatitis: G2	14.02	10.40	13.23	9.45	11.78
Diarrhea: G2	11.89	20.80	13.54	15.24	15.37
Diarrhea: G3	6.71	15.90	6.77	15.55	11.23
Dizziness: G2	0.61	2.45	0.92	2.74	1.68
Dysgeusia: G2	0.00	1.83	0.61	1.83	1.07
Fatigue: G2	14.33	24.46	19.08	21.95	19.96
Fatigue: G3	1.22	3.98	2.15	5.79	3.29
Fever: G2	0.92	2.45	0.31	3.96	1.91
Hemorrhoids: G2	0.92	0.92	1.85	3.66	1.83
Hyperglycemia: G2	3.66	5.50	3.69	3.05	3.98
Hypertension: G2	1.22	0.61	0.61	3.35	1.45
Hypoalbuminemia: G2	1.83	3.67	0.61	2.44	2.14
Hypocalcemia: G2	0.61	2.45	0.92	2.44	1.60
Hypokalemia: G3	0.92	1.53	0.92	5.79	2.29
Hyponatremia: G3	0.61	1.83	0.00	2.13	1.14
Insomnia: G2	0.92	4.28	1.54	3.66	2.60
Nausea: G2	5.18	12.23	5.23	9.45	8.02
Nausea: G3	0.30	0.61	1.23	2.13	1.07
Palmar-plantar erythrodysesthesia syndrome: G2	0.00	0.31	2.15	2.44	1.23
Peripheral sensory neuropathy: G2	0.61	4.59	2.15	6.10	3.36
Urinary frequency: G1	1.22	2.45	1.23	3.96	2.21
Vomiting: G2	2.44	5.81	1.85	4.57	3.67
Weight loss: G2	1.83	5.50	1.23	5.49	3.51
White blood cell decreased: G2	4.27	5.50	3.69	8.23	5.42

Table S7: Percentage (%) of selected AE term:grade classes with contribution at least 0.64% and mass at least 0.96% by treatment in R04 trial

B35

Domain	Adherent		Non-adherent		Average
	Anastrozole (n = 1065)	Tamoxifen (n = 1074)	Anastrozole (n = 443)	Tamoxifen (n = 427)	
Allergy/Immunology	0.19	0.28	0.00	1.17	0.41
Cardiovascular	1.41	1.58	1.13	6.09	2.55
Constitutional Symptoms	5.82	6.24	10.16	13.58	8.95
Gastrointestinal	1.88	2.70	3.16	6.09	3.46
Neurology	2.44	1.58	6.09	7.26	4.34
Pain	11.36	8.38	27.99	15.46	15.80
Psychiatric	1.78	2.23	5.19	3.98	3.30

Table S8: Percentage (%) of selected AE domains at cycle 1 with contribution at least 5.0% by treatment in B35 trial

Domain	Adherent		Non-adherent		Average
	Anastrozole (n = 1065)	Tamoxifen (n = 1074)	Anastrozole (n = 443)	Tamoxifen (n = 427)	
Constitutional Symptoms: G2	5.73	6.05	10.16	11.94	8.47
Dermatology/ Skin: G2	2.82	2.42	4.51	3.28	3.26
Endocrine: G2	22.63	26.63	27.31	31.85	27.11
Gastrointestinal: G2	1.69	2.61	2.94	5.15	3.1
Neurology: G2	1.97	1.4	5.19	5.39	3.49
Other: G2	2.16	2.05	2.94	5.15	3.07
Pain: G2	10.8	7.63	23.25	11.71	13.35
Pain: G3	0.66	0.84	5.64	4.21	2.84
Psychiatric: G2	1.69	1.86	4.51	2.58	2.66
Sexual/Reproductive Function: G2	4.13	2.7	5.64	3.9	4.11

Table S9: Percentage (%) of selected AE domain:grade classes at cycle 1 with contribution at least 1.64% by treatment in B35 trial

Term	Adherent		Non-adherent		Average
	Anastrozole (n = 1065)	Tamoxifen (n = 1074)	Anastrozole (n = 443)	Tamoxifen (n = 427)	
Arthralgia	7.04	4.66	18.51	9.6	9.95
Bone pain	1.6	0.84	4.74	1.64	2.2
Constipation	0.19	1.12	0.68	1.87	0.96
Dizziness/ lightheadedness	0.56	0.47	1.35	3.28	1.42
Dyspnea	1.03	1.58	2.26	3.75	2.15
Edema	0.66	0.84	0.45	1.87	0.95
Fatigue	3.29	2.61	7.45	6.79	5.03
Headache	0.75	1.02	3.84	1.64	1.81
Hot flashes/flushes	22.54	26.54	27.09	31.62	26.94
Insomnia	1.13	0.65	2.03	1.87	1.42
Mood alteration/ depression	1.41	1.86	4.29	3.75	2.83
Myalgia	3.29	2.33	7.45	4.68	4.44
Radiation dermatitis	1.22	0.56	0.9	1.64	1.08
Rash/desquamation	2.16	2.33	3.84	2.34	2.67
Sweating	2.07	2.89	3.16	6.09	3.55
Vaginal dryness	3.85	1.96	4.97	2.34	3.28

Table S10: Percentage (%) of selected AE terms at cycle 1 with contribution at least 0.94% and mass at least 0.47% by treatment in B35 trial

Term:Grade	Adherent		Non-adherent		Average
	Anastrozole (n = 1065)	Tamoxifen (n = 1074)	Anastrozole (n = 443)	Tamoxifen (n = 427)	
Arthralgia: G2	6.761	4.38	15.12	7.73	8.5
Arthralgia: G3	0.282	0.28	3.61	1.64	1.45
Bone pain: G2	1.596	0.74	4.29	0.94	1.89
Chest pain: G2	0.282	0.47	1.35	0.94	0.76
Constipation: G2	0.188	1.12	0.68	1.87	0.96
Dizziness/ lightheadedness: G2	0.563	0.37	1.13	2.34	1.1
Dyspareunia: G2	0.751	0.19	1.13	0.7	0.69
Dyspnea: G2	0.939	1.4	2.03	1.87	1.56
Dyspnea: G3	0.188	0.19	0.23	1.64	0.56
Edema: G2	0.657	0.84	0.45	1.64	0.9
Fatigue: G2	3.192	2.42	7.45	5.39	4.61
Headache: G2	0.469	1.02	3.61	0.94	1.51
Hot flashes/ flushes: G2	22.535	26.54	27.09	31.62	26.94
Infection without neutropenia: G3	0.469	0.84	0	0.94	0.56
Insomnia: G2	0.939	0.65	1.81	1.87	1.32
Mood alteration-depression: G2	1.315	1.68	3.61	2.34	2.24
Mood alteration-depression: G3	0.094	0.19	0.45	1.4	0.53
Myalgia: G2	3.192	2.14	5.64	3.51	3.62
Myalgia : G3	0.094	0.19	1.81	1.17	0.81
Nausea: G2	0.376	0.19	1.13	1.17	0.71
Neuropathy-sensory: G2	0.376	0.19	2.03	0.23	0.71
Sweating: G2	2.066	2.89	3.16	6.09	3.55
Vaginal dryness: G2	3.85	1.96	4.97	2.34	3.28

Table S11: Percentage (%) of selected AE term:grade classes at cycle 1 with contribution and mass at least 0.52% by treatment in B35 trial
